# Development and verification of an immune‐related gene pairs prognostic signature in ovarian cancer

**DOI:** 10.1111/jcmm.16327

**Published:** 2021-02-04

**Authors:** Bao Zhang, Xiaocui Nie, Xinxin Miao, Shuo Wang, Jing Li, Shengke Wang

**Affiliations:** ^1^ Department of Obstetrics and Gynecology Shengjing Hospital of China Medical University Shenyang China; ^2^ Department of Obstetrics and Gynecology Shenyang women's and children's hospital Shenyang China

**Keywords:** bioinformatics, immune‐related gene pairs, ovarian cancer, prognostic signature, TCGA

## Abstract

Ovarian cancer (OV) is the most common gynaecological cancer worldwide. Immunotherapy has recently been proven to be an effective treatment strategy. The work here attempts to produce a prognostic immune‐related gene pair (IRGP) signature to estimate OV patient survival. The Gene Expression Omnibus (GEO) and Cancer Genome Atlas (TCGA) databases provided the genetic expression profiles and clinical data of OV patients. Based on the InnateDB database and the least absolute shrinkage and selection operator (LASSO) regression model, we first identified a 17‐IRGP signature associated with survival. The average area under the curve (AUC) values of the training, validation, and all TCGA sets were 0.869, 0.712, and 0.778, respectively. The 17‐IRGP signature noticeably split patients into high‐ and low‐risk groups with different prognostic outcomes. As suggested by a functional study, some biological pathways, including the Toll‐like receptor and chemokine signalling pathways, were significantly negatively correlated with risk scores; however, pathways such as the p53 and apoptosis signalling pathways had a positive correlation. Moreover, tumour stage III, IV, grade G1/G2, and G3/G4 samples had significant differences in risk scores. In conclusion, an effective 17‐IRGP signature was produced to predict prognostic outcomes in OV, providing new insights into immunological biomarkers.

## 1 INTRODUCTION

Ovarian cancer (OV) is the most common gynaecological cancer and is a major cause of cancer‐related death among females worldwide. The number of new cases was estimated at approximately 295 414, and the number of deaths was approximately 184 799 in 2018.^1^ A family history of OV is an important risk factor with known genetic predisposition.^2^ Standard treatments include platinum‐based chemotherapy, but most tumours become resistant to the treatment.^3^ Despite improved treatment outcomes in recent years, the prognosis of patients with advanced OV remains poor. The emergence of chemoresistant diseases confined to the peritoneum is the leading cause of death.^4^ Thus, an in‐depth understanding of the molecular functions of OV could lead to new diagnostic, predictive, prognostic and therapeutic biomarkers.

Substantial interest in the field of immunotherapy has recently emerged, and immunotherapy has been proven effective in the treatment of human malignancies.^5^ Altered phenotype and function of major immune cell subsets (including bone marrow cells, macrophages, dendritic cells and T cells) in the OV microenvironment have been reported in response to immunotherapy.^6^ Pre‐clinical studies have also been conducted in the past decade, with most emphasis on the use of set protein 1 (PD‐1) or its ligand (PD‐L1) to induce cell death. Research based on tumour biology is exploring new and more effective immunotherapies. There have been several combination therapies, such as checkpoint inhibitors, anti‐VEGF therapy, PARP inhibitors and adoptive immunotherapies in OV treatments.^7^ Additionally, targeting other immunosuppressive pathways may become a way to enhance responses to immunotherapy.

Recently, based on microarray and RNA‐sequencing methods, there have been increasing studies on immune‐related prognostic signatures in human cancers. For example, using a cohort of glioma samples with expression information from whole‐genome microarrays from the Chinese Glioma Genome Atlas and TCGA databases, researchers constructed a local risk signature associated with immunity that can independently identify patients with a high risk.^8^ Wang *et al* ^9^ used the TCGA and ImmPort databases to build a 15‐gene prognostic model in renal papillary cell carcinoma. Their signature was associated with tumour staging and tumour type. Other immune‐related signatures have also been reported in cancers, including gastric cancer,^10^ anaplastic gliomas,^11^ breast cancer^12^ and pancreatic cancer.^13^ However, there have been no reports of immune‐related gene signatures in OV.

In the present study, we used the genetic expression profiles and clinical data of OV cases harvested according to the TCGA and GEO databases, which were divided into training, validation and testing sets. Based on the InnateDB database and LASSO regression model, we identified a 17‐IRGP signature that was significantly associated with survival. When compared with four other signatures in OV, our 17‐IRGP signature had better prognostic prediction performance. In conclusion, an effective 17‐IRGP signature was produced to predict prognostic outcome in OV, providing new insights into immunological biomarkers.

## 2 METHODS

### 2.1 Gene expression data source

The OV datasets in this study were derived from the TCGA^14^ and GEO^15^ databases. First, we used the GDC Data Transfer Tool to download RNA‐sequencing data as FPKM files and corresponding clinical information of patients from the TCGA (https://portal.gdc.cancer.gov/) database. The download time was March 2019. Here, a total of 334 OV samples were included. Second, the GEO database (https://www.ncbi.nlm.nih.gov/geo/) provided OV gene expression profiles, including GSE14764 (n = 80 samples) ^16^ and GSE26712 (n = 195 samples).^17^ They were both performed on the GPL96 platform (Affymetrix Human Genome U133A Array).

### 2.2 Data processing

To ensure the analysis consistency in different datasets, we downloaded the raw data of GSE14764 and GSE26712 and used the robust multiarray average (RMA) method^18^ for homogenization. Because both GEO datasets were performed by GPL96 platform, we merged them into an independent external validation set for subsequent analysis and performed batch correction to eliminate batch effects. Before constructing the prognostic signature, the original data were pre‐processed by 1) removing tumour samples without clinical information and overall survival (OS) was 0 day; 2) removing normal samples; 3) removing genes with low expression (gene expression was missing or 0 in more than half of all samples); and 4) retaining only the expression profiles of immune‐related genes. The clinical information of patients in two GEO datasets was shown in Table S7. The pre‐processed dataset ultimately contained a total of 594 OV samples.

### 2.3 Computation of immune‐related gene pairs

First, we downloaded the genes associated with immune from the InnateDB dbase (https://www.innatedb.com/).^19^ This database records the innate immune‐related genes of multiple species that are supported by the literature and manually corrected. Here, we obtained endogenous human IRGs. After sorting (removing genes with duplicate symbols), there were a total of 1039 IRGs (Table [Link], [Link]). IRGPs were constructed based on 1,039 IRGs with the calculation rules as described previously.^20^ In brief:

If Expr*_IRG1_* < Expr*_IRG2_*, IRGP = 1, else: IRGP = 0.

Expr*_IRG1_* is the expression value of immune gene 1, and Expr*_IRG2_* is the expression value of immune gene 2. According to the above rules, the IRGP value of each dataset was calculated separately, and a total of (1,039 * 1,038)/2 IRGPs were obtained. After removing samples in all datasets with a gene pair value of 0 or 1, we left the residual IRGPs and subsequently selected them as first candidate IRGPs to conduct further analysis (Figure [Link], [Link]).

### 2.4 Construction of prognostic IRGP signatures

First, 334 samples were divided into the training or validation set in the TCGA dataset. To avoid the influence of random assignment bias on the stability of the subsequent modelling, all samples were put back into random groupings 100 times. The samples were selected according to a ratio of 1:1 in the training set and validation set. Here, the training set and validation set with no significant differences in the distributions of age, tumour stage, follow‐up time and patient survival status were selected for signature construction. The resulting training set and validation set samples are available in Table 1. The prognostic signature was constructed in two steps as follows: 1) a univariate Cox proportional hazards regression model was employed for the calculation of the relationship between each IRGP and patients’ prognosis with log‐rank test *P*‐value < 0.05; 2) we used the LASSO regression to further filter the above IRGPs to reduce the numbers in the risk model.

**TABLE 1:** The clinical information distributions of training set and validation set samples

Clinical features	Training set (n = 166)	Validation set (n = 166)	Chi‐squared test *P*‐value
Status			
Alive	69	62	0.5005
Dead	97	104	
Grade			
G1_G2	21	21	1
G3_G4	140	143	
GX	5	2	
Stage			
II	11	11	0.8261
III	127	131	
IV	27	23	
Unknown	1	1	
Age			
0 ~ 50	33	35	0.9278
50 ~ 60	56	51	
60 ~ 70	42	39	
70 ~ 80	29	34	
80 ~ 100	6	7	

Note

GX represents grade cannot be assessed.

### 2.5 Functional enrichment analysis

We used the clusterProfiler R package^21^ to analyse the IRGPs for molecular function (MF), cellular component (CC), biology procedure (BP) enrichment by studying the Gene Ontology (GO) terms. A q‐value <0.05 was set as the threshold for significant enrichment. The dot plot of clusterProfiler displayed the enrichment results. We took the genes with differential expression (DEGs) in groups of high and low risk with the use of the rank test with false discovery rate (FDR) < 0.05, and the Kyoto Encyclopedia of Genes and Genomes (KEGG) pathway enrichment of DEGs was carried out using the GSVA R package.

### 2.6 Evaluation of IRGP prognostic signature

Based on the 17 IRGPs obtained by LASSO regression and its regression coefficient, we obtained the risk score of each respective OV patient by:

Risk score = ∑IRGP * coefficient

Here, the IRGP was the immune gene pair, and the coefficient was the regression coefficient. We split all OV samples into a low‐risk group (Risk‐L) or a high‐risk group (Risk‐H) in line with the median risk score. We used the performance of this signature to plot the receiver operating characteristic (ROC) curves.

### 2.7 Comparison of IRGP risk signatures with other models

To evaluate the performance of the 17‐IRGP risk model and other existing prognostic models, we selected models that were also constructed based on gene expression data for comparison. After consulting the literature, four prognostic risk models were selected: Liu (7‐gene signature, 2018, based on GSE32062 for Japan cohort in GEO, GSE63885 for Poland cohort in GEO, and E‐MTAB‐386 for USA cohort in arrayexpress),^22^ Liu (5‐gene signature, 2016, based on TCGA database),^23^ Hou (6‐gene signature, 2018, based on n TCGA database with records of Taxol treatment),^24^ and Zhang (2‐gene signature, 2018, based on TCGA database).^25^ To make the model more comparable, we used the same method for the calculation of the risk score of each OV sample and evaluated the AUC value of each model. According to the median risk score, the samples were also split into Risk‐H and Risk‐L groups, and the difference in overall survival between the two groups was calculated by log‐rank test. Using the concordance index (C‐index) and restricted mean survival (RMS), we assessed the prognosis precision of the signature.^26^ RMS can be interpreted as the average free‐event survival time in a specific time period, which is equivalent to the area under the Kaplan‐Meier (K‐M) survival curves at a specific time‐point. The higher the RMS time ratio, the greater the difference is in prognosis. Here, we evaluated the period between 0 and 150 months RMS of each model and drew the RMS curve.

### 2.8 Statistical analysis

With the use of R (version 3.6.1) software, we carried out statistical analyses. We used the survival R package for univariate and multivariate risk regression analysis, the glmnet package for LASSO Cox regression model, and the survivalROC package to evaluate the model's ROC curve and calculated AUC values. Moreover, the KMsurv R package was performed to show the K‐M curves of grouped samples. The C‐index calculation used the survcomp R package, and RMS cure and RMS time calculation were performed by using the survival and survRM2 packages. In terms of each test, a p‐value < 0.05 suggested a significant difference. *P‐value <0.05, ** p‐value < 0.01, and ***P‐value <0.001 express statistically significant characteristics.

## 3 RESULTS

### 3.1 Construction of IRGP signatures

**FIGURE 1:**
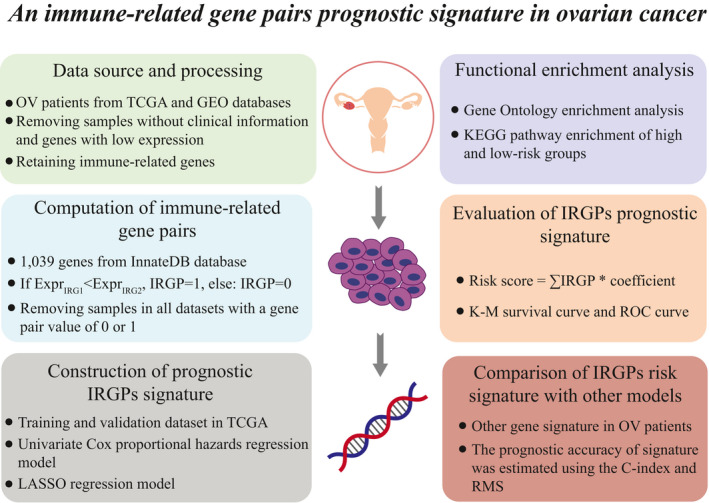
The workflow of this study. Production and verification of a gene pair prognostic signature related to immunity in ovarian cancer

In this study, in accordance with the gene expression profiles of OV from the TCGA database, we performed a series of bioinformatics analyses to build a model for prognostic evaluation of IRGPs in OV patients (Figure 1). Here, a total of 1039 IRGs from the InnateDB database were obtained. Of these genes, 539 241 IRGPs were established. After removing samples in all datasets with gene pair values of 0 or 1, we left the residual IRGPs and subsequently selected them as first candidate IRGPs to conduct further analysis. Then, a univariate Cox proportional hazards regression model was adopted for the calculation of the relationship between each IRGP and patient survival with a log‐rank test *P*‐value <0.05. A total of 3,765 identified IRGPs were identified (Table [Link], [Link]). Moreover, using the LASSO regression model, we further selected 17 IRGPs for the final risk prognostic signature (Table 2, Figure [Link], [Link]).

**TABLE 2:** The results of 17 IRGPs using LASSO regression model

IRGPs	Coef	*P*‐value	HR	Low.95CI	High.95CI
LILRA2 vs P2RY14	1.083615	0.00014	2.955345	1.69188	5.162343
NOD2 vs LILRA2	−1.15639	0.000278	0.31462	0.168658	0.586905
CXCL14 vs SHARPIN	0.877286	0.003049	2.404364	1.345709	4.295853
TSC22D3_vs_CXCL11	1.177209	0.005145	3.245304	1.422665	7.403006
FOXA2_vs_RCAN1	−0.68767	0.007236	0.502744	0.30437	0.83041
PLA2G4A vs SCAF11	−0.84326	0.019819	0.430307	0.211683	0.874725
ABCA1 vs MST1R	0.75507	0.027164	2.127761	1.088905	4.157725
STAT4 vs IL1R2	−0.85268	0.031899	0.426269	0.195622	0.928859
AP3B1 vs BTN3A3	0.525508	0.040716	1.691318	1.022447	2.797755
MID1 vs THBS1	−1.69541	0.069621	0.183524	0.029397	1.145736
IFNGR1 vs CASP6	0.483861	0.250733	1.622325	0.710478	3.704465
MSR1 vs CXCL11	−0.33671	0.265347	0.714119	0.394875	1.291463
BTN3A2 vs IRF2	−0.68764	0.281319	0.502761	0.143903	1.756516
IL1B vs CXCR3	0.37003	0.284697	1.447778	0.735004	2.851768
SNX27 vs CXCL11	0.364458	0.388464	1.439734	0.628867	3.296136
CASP7 vs CXCL11	0.274334	0.509042	1.315654	0.582788	2.970113
BTN3A3 vs TPST1	−0.00035	0.998939	0.999646	0.59306	1.684976

Abbreviations: Coef, coefficient by LASSO analysis; HR, hazard ratio; High.95CI, high 95% CI; Low.95CI, low 95% confidence interval (CI).

### 3.2 The evaluation and validation of IRGP signatures for survival prediction

**FIGURE 2:**
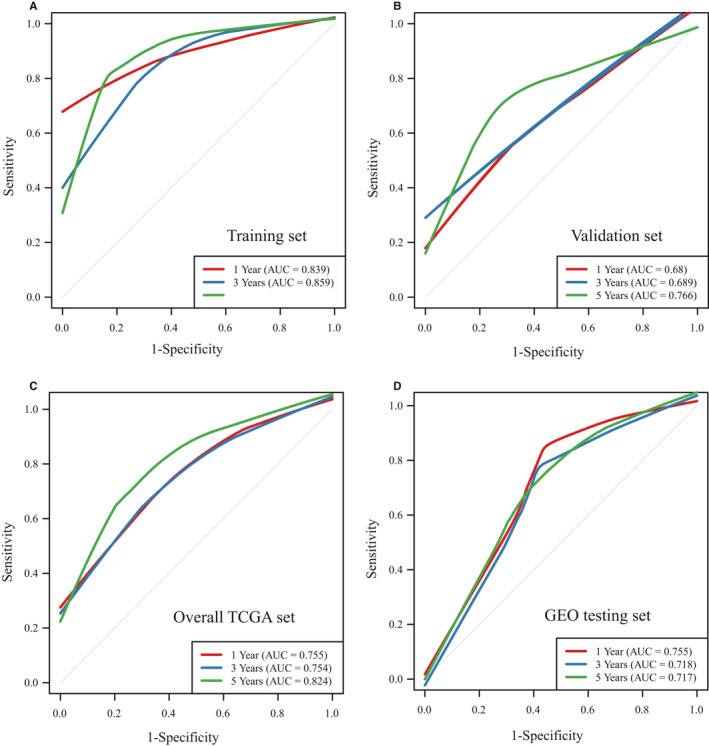
The time‐dependent ROC curve of OV patients based on the IRGPs. A, Training set. B, Validation set. C, Overall TCGA set. D, GEO testing set

**FIGURE 3:**
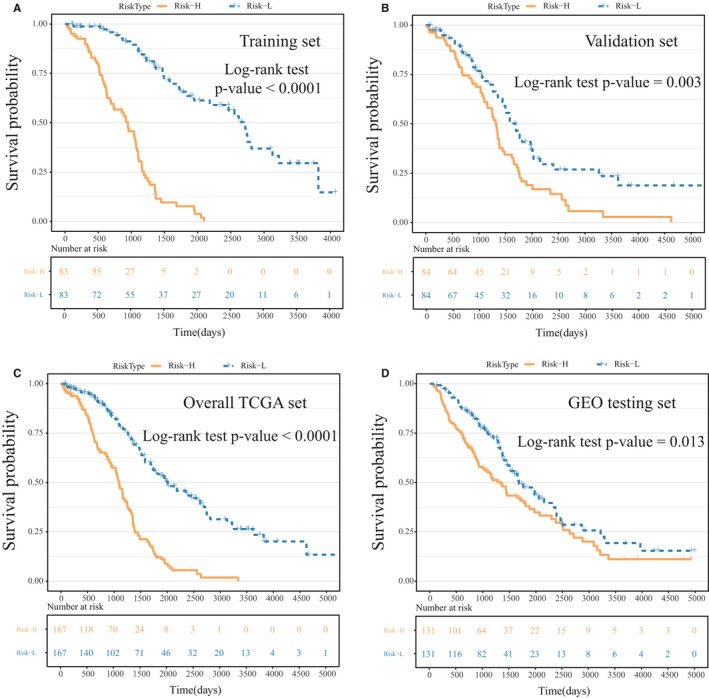
The Kaplan‐Meier curves of total survival of various IRGP signature risk groups. OV cases were stratified by median risk scores (Risk‐H and Risk‐L groups). A, Training set. B, Validation set. C, Overall TCGA set. D, GEO testing set

Based on the above 17 IRGPs, we constructed a prognostic risk model for OV patients. Since the overall survival time of patients was distributed over more than 2 years (Figure [Link], [Link]), the predictive effect of this model on datasets for 1, 3 and 5 years was evaluated. Next, the IRGPs were adopted for the calculation of the risk score for the respective case in the TCGA training group. The average AUC of the training set was 0.869, and the average AUC of the validation set was 0.712 (Table [Link], [Link]). In addition, the average AUC of all TCGA datasets was 0.778, and the average AUC of the independent testing set was 0.73 (Figure 2A‐D). By dividing OV patients into low‐ (Risk‐L) and high‐risk groups (Risk‐H) based on the median risk score, we observed that the Risk‐H group exhibited noticeably poorer prognosis than the Risk‐L group on the training set, validation set, all TCGA set and independent GEO testing set (log‐rank test *P*‐value <0.05, Figure 3A‐D).

### 3.3 Functional analysis of immune‐related gene pairs

**FIGURE 4:**
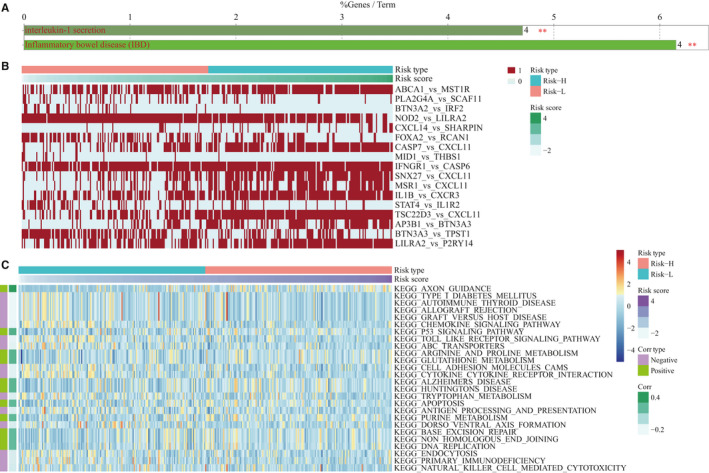
Functional analysis of 29 immune‐related genes. A, Gene family enrichment results for 29 immune‐related genes. B, Relationship between 17 IRGPs values and risk scores. C, GSVA pathway enrichment results for DEGs in high‐ and low‐risk groups. Corr represents the correlation coefficient between the enrichment scores and the sample risk scores with FDR < 0.05

In our signature, the 17 IRGPs contained a total of 29 immune genes. These genes were significantly enriched in the ‘caspase’ and ‘interferon receptor’ families (*P*‐value <0.01, Table 3). The GO annotation results of 29 genes suggested that 4 genes were significantly enriched in interleukin‐1 secretion and inflammatory bowel disease biological processes (Figure 4A). Seven of the 17 IRGPs showed a negative correlation with risk scores, and ten showed a positive correlation (Figure 4B, Table 4). Here, IRGPs reflected the level of relative expression between two genes. We found that genes related to immune activation/response (P2RY14, CXCL11, CXCR3, MST1R and NOD2) showed relatively low expression levels (Table [Link], [Link]). The relatively highly expressed genes (CASP6, CASP7, IL1B, THBS1 and TPST1) were mainly related to the apoptotic process and inflammation response (Table [Link], [Link]). Further enrichment analysis of DEGs in the high‐ and low‐risk groups was performed (Table [Link], [Link]). The results suggested that the immune response‐related pathways (Toll‐like receptor and chemokine signal pathway, and others) were significantly negatively correlated with risk scores; however, the pathways such as p53 signalling pathway and apoptosis had a positive correlation with the risk scores (Figure 4C), which seems to indicate that samples from the low‐risk group may have higher immune activity (or immune activation status).

**TABLE 3:** The gene family enrichment results of 29 immune‐related genes

Gene family	Genes	*P*‐value	FDR
Caspases	CASP7/CASP6	0.000168	0.005042
Interferon receptors	IFNGR1	0.007737	0.232112
P2Y receptors	P2RY14	0.011584	0.347516
V‐set domain containing	BTN3A2/BTN3A3	0.019208	0.576234
ATP binding cassette subfamily A	ABCA1	0.019234	0.577029
Clathrin/coatomer adaptor, adaptin‐like, N‐terminal domain containing	AP3B1	0.019234	0.577029
Zinc fingers RANBP2‐type	SHARPIN	0.028087	0.842623
NLR family	NOD2	0.033112	0.993349
Scavenger receptors	MSR1	0.035614	1
Sorting nexins	SNX27	0.038111	1
Sulfotransferases, membrane bound	TPST1	0.048034	1
Receptor tyrosine kinases	MST1R	0.05173	1
Phospholipases	PLA2G4A	0.054186	1
Interleukin receptors	IL1R2	0.054186	1
Interleukins	IL1B	0.055412	1
Forkhead boxes	FOXA2	0.055412	1
Chemokine ligands	CXCL14	0.057858	1
CD molecules	LILRA2/CXCR3	0.092531	1
Tripartite motif containing	MID1	0.117076	1
SH2 domain containing	STAT4	0.123936	1
Endogenous ligands	CXCL11	0.258581	1
Ring finger proteins	SCAF11	0.327935	1
Unknown	IRF2:RCAN1:THBS1:TSC22D3	1	1

**TABLE 4:** Analysis of correlation between gene pair values and risk scores

IRGPs	*P*‐value	Pearson correlation	Type
TSC22D3_vs_CXCL11	1.08E‐27	0.54906008	Positive
CASP7_vs_CXCL11	4.08E‐23	0.50599649	Positive
SNX27_vs_CXCL11	1.42E‐19	0.467906634	Positive
AP3B1_vs_BTN3A3	5.90E‐16	0.42334027	Positive
IL1B_vs_CXCR3	6.39E‐14	0.39504485	Positive
MSR1_vs_CXCL11	1.10E‐12	0.37640839	Positive
LILRA2_vs_P2RY14	2.03E‐12	0.372271702	Positive
ABCA1_vs_MST1R	9.10E‐10	0.32707845	Positive
CXCL14_vs_SHARPIN	9.39E‐07	0.264532336	Positive
IFNGR1_vs_CASP6	2.01E‐03	0.168462127	Positive
PLA2G4A_vs_SCAF11	2.69E‐04	−0.198123738	Negative
MID1_vs_THBS1	1.16E‐08	−0.305853734	Negative
FOXA2_vs_RCAN1	3.98E‐11	−0.3511514	Negative
BTN3A3_vs_TPST1	3.63E‐11	−0.35182302	Negative
STAT4_vs_IL1R2	1.72E‐11	−0.357242428	Negative
BTN3A2_vs_IRF2	1.73E‐12	−0.37335433	Negative
NOD2_vs_LILRA2	1.09E‐12	−0.376498688	Negative

### 3.4 Relationship between prognostic risk signature and clinical features

**FIGURE 5:**
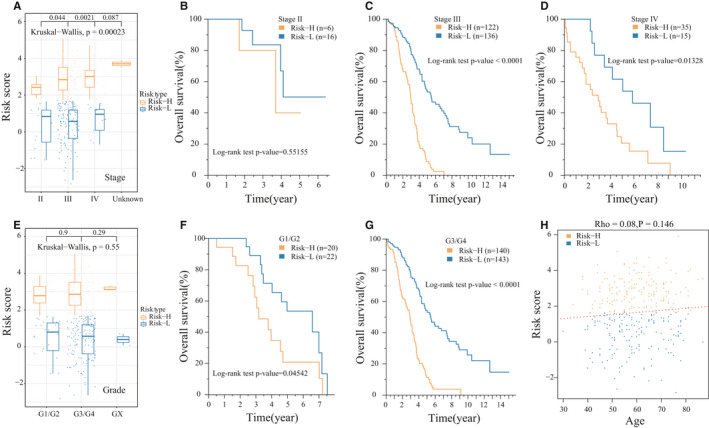
Relationship between the 17‐IRGP risk model and clinical characteristics. A, Stage risk distribution. B, The K‐M curve of stage II of high‐ and low‐risk samples. C, The K‐M curve of stage III high‐ and low‐risk samples. D, The K‐M curve of stage IV of high‐ and low‐risk samples. E, The grade risk distribution. F, The K‐M curve of G1/G2 of high‐ and low‐risk samples. G, The K‐M curve of G3/G4 of high‐ and low‐risk samples. H, The correlation between age and risk scores

Using clinical information such as age, tumour stage and grade from the TCGA database, we analysed the relationship between the 17‐IRGP risk signature and clinical characteristics. Here, samples from the tumour stage II, III and IV groups showed significant differences in risk scores (Figure 5A), but no significant difference was observed for prognosis of the high‐ and low‐risk group samples in stage II (*P*‐value >0.05). However, significant differences were shown in stages III and IV, indicating that the model may be more suitable for stage III/IV OV patients (Figure 5B‐D). For tumour grades, G1/G2 and G3/G4 samples had no significant difference in risk scores (Figure 5E). However, the prognosis of the high‐ and low‐risk group samples showed significant differences in G1/G2 and G3/G4 (Figure 5F‐G). We also did not observe a significant correlation between age and risk scores (Figure 5H).

### 3.5 The performance of prognostic risk signature in OV subtypes

**FIGURE 6:**
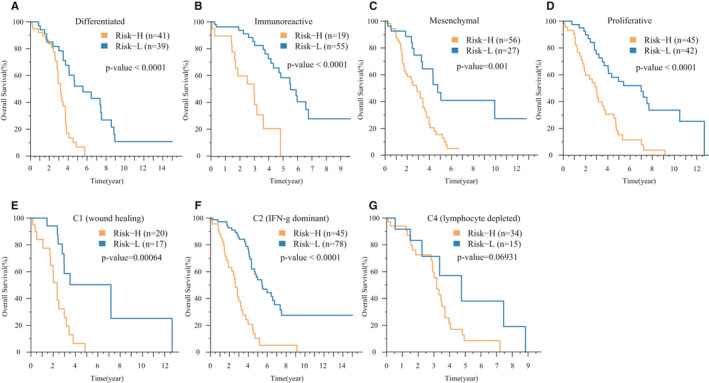
The performance of prognostic risk signature in OV subtypes. A‐D, The K‐M curves of the 17‐IRGP risk model on differentiated, immunopositive, mesenchymal and proliferative subtypes of the TCGA database. E‐G, The K‐M curves of the 17‐IRGP risk model on immune subtypes C1, C2 and C4

The TCGA project revealed that surviving gene expression characteristics can predict clinical outcomes and divide OV patients into four transcription subtypes, including differentiated, immunoreactive, mesenchymal and proliferative.^27^ We next compared the prognostic performance of our model on these four molecular subtypes. Low‐ and high‐risk groups of the four subtypes were identified to have significant prognostic differences (Figure 6A‐D). In addition, we also found the best prognosis in immunoreactive subtype, Risk‐L samples, while the worst prognosis mesenchymal subtype, Risk‐H samples. Moreover, OV was divided into four immune subtypes (C1‐C4)^28^ based on immune molecular tags. We further compared the model's performance on different immune subtypes (the C3 immune subtype had only 3 samples and was not added to the analysis). Among the above three immune subtypes, there were also different survival outcomes between the high‐ and low‐risk groups in both the C1 and C2 immune subtypes (Figure 6E‐G).

### 3.6 Comparison of our prognostic risk signature with other models

**FIGURE 7:**
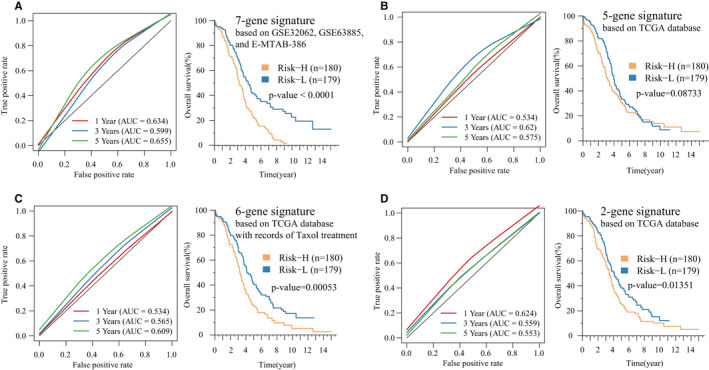
The ROC and K‐M curves of the OV prognostic risk model. A, The ROC and K‐M curve of Risk‐H/Risk‐L samples of the 7‐gene signature risk model. B, The ROC and K‐M curve of Risk‐H/Risk‐L samples of the 5‐gene signature risk model. C, The ROC and K‐M curve of Risk‐H/Risk‐L samples of the 6‐gene signature risk model. D, The ROC and K‐M curve of Risk‐H/Risk‐L samples of the 2‐gene signature risk model

**FIGURE 8:**
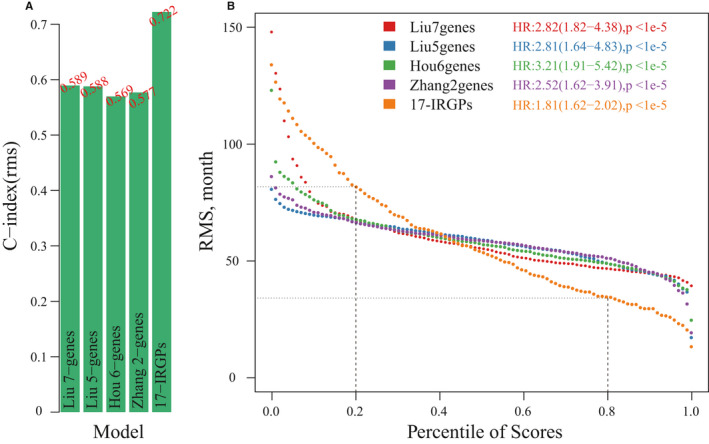
Comparison of OV prognostic risk models. A, The C‐index of 5 prognostic risk models. B, The RMST curves of 5 prognostic risk models. The dashed line represents the RMS time (months) corresponding to the 20th and 80th percentile scores. Each point represents the corresponding model's RMS time corresponding to the sample risk score

Finally, using the same method, we evaluated the AUC values of four existing OV prognostic models at 1, 3 and 5 years. The average AUC values of these 4 models were all < 0.7, which were lower than the AUC of our 17‐IRGPs signature, indicating that our model has better prediction performance. Among the 4 models, only the Risk‐H and Risk‐L groups calculated by the 5‐gene signature model have no significant difference in prognosis, and other 3 models showed significant differences in prognosis (Figure 7A‐D). Based on the C‐index of above five prognostic models, the 17‐IRGPs model has the largest C‐index (Figure 8A), indicating that the overall performance of our model was better than the other four models. The RMST curves of the five models also show significant differences. The risk scores of these models have a very significant relationship with the prognosis (HR > 1, *P*‐value <0.0001), but we see that the RMST cure of 17‐IRGPs was better than the other four models, which has a steeper slope (Figure 8B), indicating that our model can better evaluate the survival rate in OV.

Moreover, we examined using GSE14764 and GSE26712, separately. As shown in Figure S4, we first used the same method in the GSE14764 dataset to evaluate the AUC values of four existing OV prognostic models. We can see that AUC values of all four existing OV prognostic models are very small. The results of most K‐M curves are not statistically significant. Similar results can still be revealed in GSE26712 dataset (Figure S5). Therefore, combining the above analysis results, we can find that in TCGA, GSE14764 or GSE26712 dataset, the AUC values of four existing OV prognostic models are very small, indicating that our 17‐IRGPs model can better evaluate the survival rate in OV (Figure S6).

## 4 DISCUSSION

In this study, based on the TCGA database and LASSO regression model, we identified a 17‐IRGP signature that was significantly associated with survival. This robust 17‐IRGP signature can estimate prognosis in OV and provide new insights into immunological biomarkers.

Immunotherapy strategies in cancers aim to develop combination methods to enhance immunity and prevent local immunosuppression. Chimeric antigen receptor‐modified T cells, cancer vaccines, immune checkpoint blockade and antibody‐based therapies have shown pre‐clinical success and have been clinically tested in OV.^29^ Supported by the methods of RNA‐sequencing and microarray, as well as available gene expression databases such as TCGA and GEO, an increasing number of reports of gene prognosis models of cancers have appeared recently. For example, studies have been reported in melanoma,^30^ breast cancer,^31^ clear cell renal cell carcinoma ^32^ and other cancers. However, there are few reports on immune‐related gene signature associated with cancer prognosis. In colorectal cancer (CRC), Wu *et al* ^33^ used genetic expression profiles and clinical data of cases to construct a 19‐IRGP signature that covers 36 individual genes. Their IRGP signature can stratify CRC cases into low‐ and high‐risk groups by prognostic outcome. This effective IRGP signature that predicts prognostic outcomes in CRC, covering early‐stage disease, is capable of providing novel knowledge of identifying cases at a high risk of mortality. However, there is no prognostic model of IRGPs reported in OV.

Based on the functional analysis of IRGPs, the Toll‐like receptor and chemokine signal pathways were significantly negatively correlated with risk scores. Toll‐like receptors (TLRs), as the most important pattern recognition receptors in innate immunity, play an important role in inducing immune responses by recognizing microbial invaders or specific agonists.^34^ The antitumour effect of TLRs can directly induce tumour cell death and activate an effective antitumour immune response.^35^ It can trigger an inflammatory response and cell survival in the tumour microenvironment. TLR2, TLR3, TLR4 and TLR5 were reported to be highly expressed in normal and neoplastic ovarian epithelium.^36^ However, pathways such as the p53 signalling and apoptosis pathways had a positive correlation with the risk scores. Thus, the above signalling pathways were shown to be closely related to the risk scores of our signature and may be involved in the immune response to OV.

The prognostic signature in our study consists of 29 unique IRGs. CXCL11 (CXC chemokine ligand 11) is a chemokine involved in the progression of various cancers. CXCL11 is overexpressed in CRC tissues and cell lines. Repression of CXCL11 significantly inhibited cell migration, invasion and epithelial‐mesenchymal transition (EMT).^37^ It was also reported that its down‐regulation can inhibit tumour angiogenesis in epithelial OV.^38^ High CXCL11 expression was determined to predict worse OS in high‐grade serous OV.^39^ STAT4 (signal transducer and activator of transcription 4) is a member of the STAT family. Its overexpression was shown to be associated with poor prognosis in OV patients.^40^ It has also been reported to be involved in the occurrence and development of gastric cancer ^41^ and hepatocellular cancer.^42^ In addition, the expression of forkhead box A2 (FOXA2) in colon cancer tissues is up‐regulated and related to the metabolism and clinical stages.^43^ Moreover, FOXA2 is capable of facilitating EMT, inhibiting apoptosis and enhancing colon cancer cell invasion ability. In OV, miR‐590‐3p can promote growth and metastasis via the FOXA2‐Versican pathway.^44^ According to the above results, the genes involved in the IRGP signature play a significant role in human cancers.

To date, many computational methods developed for cancer research have focused on identifying diagnostic or prognostic gene signatures from gene expression data that can be used as diagnostic or prognostic biomarkers. However, such gene signatures may not be found in gene expression data because gene expression levels are often sensitive to systematic bias measurements.^45^ Gene pairs are more reliable prognostic markers than single genes because they can be found even in gene expression profiling where no significant prognostic genes are present.^46^, ^47^ In our study, we identified an effective 17‐IRGP signature for OV patients, which have a better prognostic assessment ability than .

There are also some limitations to our study. First, the robustness of IRGPs was based on the gene expression profiles produced by RNA‐sequencing and microarray data and must be verified in large clinical samples of OV. Second, further experimental validation is required. Taken together, an effective 17‐IRGP signature was produced to predict prognostic outcomes in OV, providing new insights into immunological biomarkers.

## CONFLICT OF INTEREST

The authors declare no conflicts of interest.

## AUTHOR CONTRIBUTIONS

BZ and XCN designed experiments. XXM, SW and JL contributed to the literature review. BZ wrote the initial draft of the manuscript. SKW designed the study and edited the paper. All authors have approved the final version of the manuscript.

## Supporting information

Fig S1

Fig S2

Fig S3

Fig S4

Fig S5

Fig S6

Table S1

Table S2

Table S3

Table S4

Table S5

Table S6

Table S7

Table S8

Supplementary Material

## Data Availability

All data generated or analysed during this study are included in this published article and its supplementary information files.
