# Supplementary material for: Development and verification of an immune‐related gene pairs prognostic signature in ovarian cancer
Source: J Cell Mol Med. 2021 Feb 4;25(6):2918–30. doi: 10.1111/jcmm.16327 (PMC7957197; doi:10.1111/jcmm.16327)

**Supplementary Information**

**Supplementary Table Legends**

**Supplementary Table 1. The immune-related gene list from the InnateDB database.**

**Supplementary Table 2. The results of IRGPs by univariate Cox proportional hazards regression model.**

**Supplementary Table 3. The clinical information including risk scores and risk groups of all TCGA OV patients.**

**Supplementary Table 4. GO functional annotation of genes with relatively low expression.**

**Supplementary Table 5. GO functional annotation of genes with relatively high expression.**

**Supplementary Table 6. GSVA analysis of DEGs in high- and low-risk groups.**

**Supplementary Table 7. The clinical information of patients in two GEO datasets.**

**Supplementary Table 8. The comparison between this study and other gene signature studies.**

**Supplementary Figures**

**Supplementary Figure 1. The concept figure we established IRGPs and the score of each IRGP.Supplementary Figure 2. The results of the LASSO regression model.** (A) Each independent parameter selection in the LASSO model. For each independent variable, the horizontal axis represents the log value of the independent variable lambda, and the vertical axis represents the coefficient of the independent variable. (B) The confidence interval under each lambda.

**Supplementary Figure 3. The distribution of patient survival time in each dataset.** (A) TCGA dataset. (B) GEO dataset.

**Supplementary Figure 4. The ROC and K-M curves of the OV prognostic risk model in GSE14764 dataset.** (A) The ROC and K-M curve of Risk-H/Risk-L samples of the 7-gene signature risk model. (B) The ROC and K-M curve of Risk-H/Risk-L samples of the 5-gene signature risk model. (C) The ROC and K-M curve of Risk-H/Risk-L samples of the 6-gene signature risk model. (D) The ROC and K-M curve of Risk-H/Risk-L samples of the 2-gene signature risk model.

**Supplementary Figure 5. The ROC and K-M curves of the OV prognostic risk model in GSE26712 dataset.** (A) The ROC and K-M curve of Risk-H/Risk-L samples of the 7-gene signature risk model. (B) The ROC and K-M curve of Risk-H/Risk-L samples of the 5-gene signature risk model. (C) The ROC and K-M curve of Risk-H/Risk-L samples of the 6-gene signature risk model. (D) The ROC and K-M curve of Risk-H/Risk-L samples of the 2-gene signature risk model.


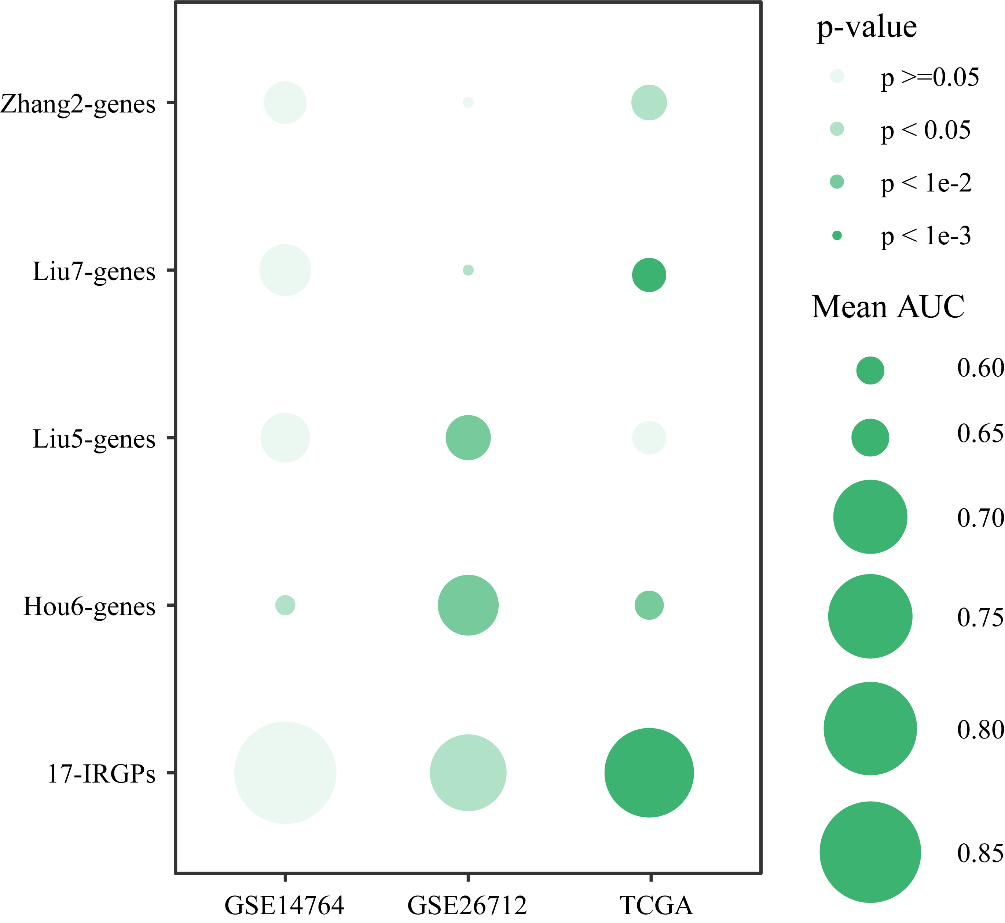


**Supplementary Figure 6. The prognostic prediction performance of four existing OV prognostic models in TCGA, GSE14764, and GSE26712 datasets.**


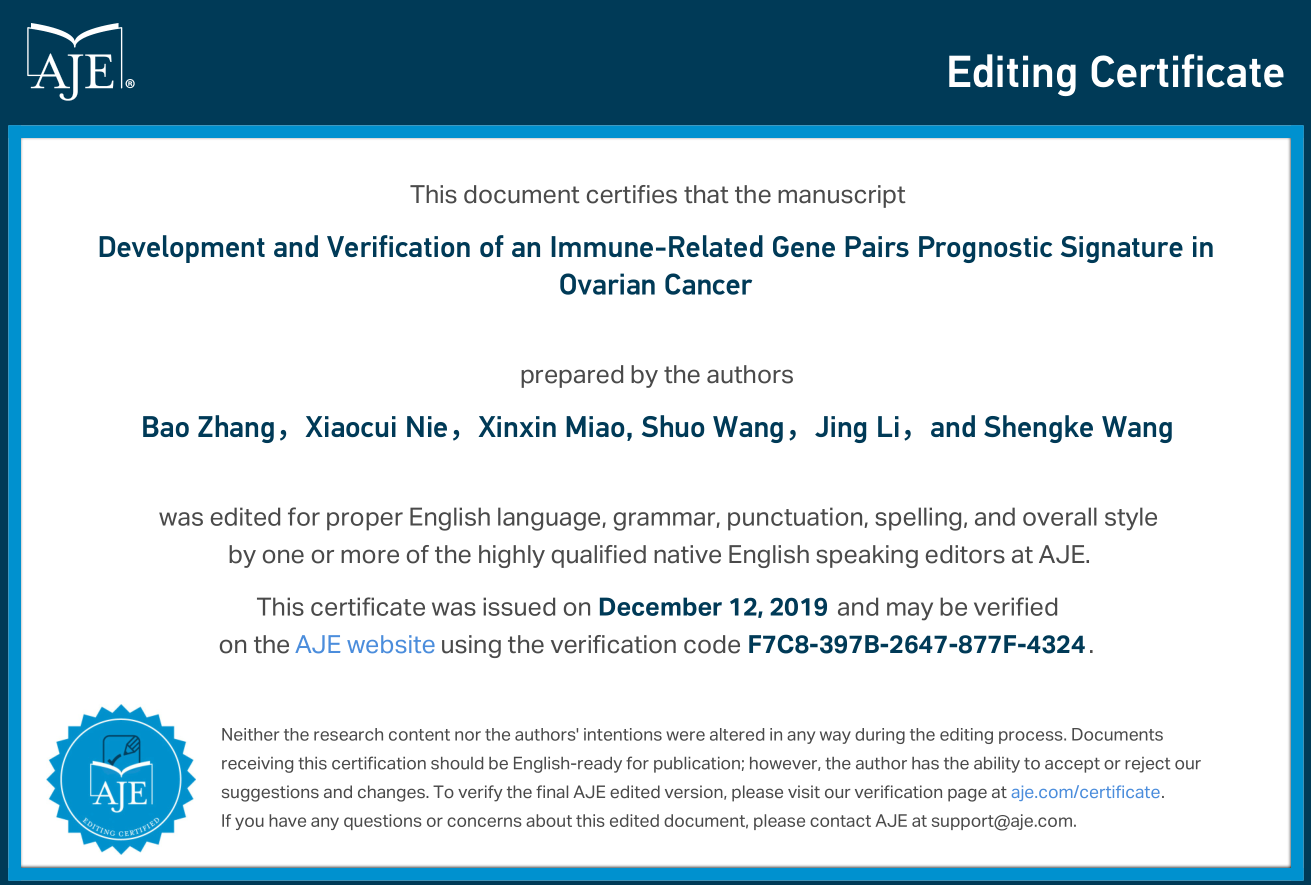

Supplement: Supplementary file 15 — Supplementary Material [file JCMM-25-2918-s003.docx]
